# Human Ischemic Cardiomyopathy Shows Cardiac Nos1 Translocation and its Increased Levels are Related to Left Ventricular Performance

**DOI:** 10.1038/srep24060

**Published:** 2016-04-04

**Authors:** Esther Roselló-Lletí, Ricardo Carnicer, Estefanía Tarazón, Ana Ortega, Carolina Gil-Cayuela, Francisca Lago, Jose Ramón González-Juanatey, Manuel Portolés, Miguel Rivera

**Affiliations:** 1 Cardiocirculatory Unit, Health Institute of La Fe University Hospital (IIS La Fe), Valencia, Spain; 2 Department of Cardiovascular Medicine, University of Oxford, United Kingdom; 3 Cellular and Molecular Cardiology Research Unit, Department of Cardiology and Institute of Biomedical Research, University Clinical Hospital, Santiago de Compostela, Spain

## Abstract

The role of nitric oxide synthase 1 (NOS1) as a major modulator of cardiac function has been extensively studied in experimental models; however, its role in human ischemic cardiomyopathy (ICM) has never been analysed. Thus, the objectives of this work are to study NOS1 and NOS-related counterparts involved in regulating physiological function of myocyte, to analyze NOS1 localisation, activity, dimerisation, and its relationship with systolic function in ICM. The study has been carried out on left ventricular tissue obtained from explanted human hearts. Here we demonstrate that the upregulation of cardiac NOS1 is not accompanied by an increase in NOS activity, due in part to the alterations found in molecules involved in the regulation of its activity. We observed partial translocation of NOS1 to the sarcolemma in ischemic hearts, and a direct relationship between its protein levels and systolic ventricular function. Our findings indicate that NOS1 may be significant in the pathophysiology of human ischemic heart disease with a preservative role in maintaining myocardial homeostasis.

Ischemic cardiomyopathy (ICM) is a major cause of heart failure (HF) and represents an enormous medical and societal burden with significant attributable morbidity and mortality^1^. Dysregulation of nitric oxide, and increased oxidative and nitrosative stress are implicated in the pathogenesis of HF^2^. Nitric oxide produced in the heart by nitric oxide synthase (NOS) is an important modulator of myocardial function^3^. Three NOS isoforms have been identified in the heart: neuronal (NOS1), inducible (NOS2), and endothelial (NOS3), all associated with calmodulin (CaM). Nitric oxide derived from NOS1 and NOS3 has important effects on contractility of cardiomyocytes. NOS1 is preferentially located in the sarcoplasmic reticulum (SR) where it is associated with the ryanodine receptor Ca^2+^ release channel (RyR). NOS3 resides in sarcolemma caveolae and in endothelial cells^4^,^5^. On the other hand, tetrahydrobiopterin (BH4) is an essential cofactor of NOS regulated by GTP cyclohydrolase 1 (GCH1) which facilitates electron transfer from NOS reductase domain, stimulates nitric oxide synthesis, and maintains and stabilises NOS dimers^6^,^7^.

The role of NOS1 as a major modulator of cardiac function and intracellular Ca^2+^ fluxes has been extensively studied in experimental models^8^,^9^. In addition to CaM, NOS1 protein and mRNA binds a number of other molecules that alter its activity or expression, such as heat shock protein 90 (HSP90), protein inhibitor of NOS1 (PIN: *DYNLL1* gene), NOS interacting protein (NOSIP), and *Mm-antiNOS1* RNA^10^,^11^,^12^. Furthermore, NOS1 negatively regulates the activity of myocardial xanthine oxidoreductase (XOR)^13^. A significant increase in NOS1 mRNA and protein expression, NOS1 translocation from the SR to the sarcolemma, has been reported in human non-ischemic dilated cardiomyopathy and animal models of HF^14^,^15^. However, NOS1 localisation, and the set of NOS-related molecules involved in the regulation of myocardial Ca^2+^ fluxes, has never been analysed in human ICM.

In this background, we used the sensitive and powerful technique of RNA sequencing (RNAseq) to identify differentially expressed genes tightly involved in this process in left ventricular (LV) tissue samples obtained from ICM patients and compared the results with non-diseased controls (CNTs). We also examined the possible alterations in NOS1 localisation, activity, and dimerisation, in addition to the potential relationship between the LV dysfunction and the protein levels of NOS1 in ICM human hearts.

## Results

### Clinical characteristics of patients

We analysed 20 ICM human hearts obtained from patients undergoing cardiac transplantation. All patients were males, with a mean age of 55 ± 8 years, and an NYHA functional classification of III–IV. Patients had previously been diagnosed with significant comorbidities, including hypercholesterolemia (15%), hypertension (33%), and diabetes mellitus (42%). Table 1 summarizes the clinical characteristics of the ICM patients. CNT samples were acquired from ten non-diseased donor hearts. The CNT group mainly consisted of males (80%), with a mean age of 47 ± 16 years.

### Gene expression analysis

Differences in transcriptome-level between ICM and CNT samples were investigated by large-scale screening of 23 heart samples (13 from ICM, 10 from CNT) using RNAseq technology. On comparing the two groups, we found 1334 differentially expressed genes, of which 649 were upregulated (≥1.5-fold increase; *P* < 0.05 for all) and 685 were downregulated (≥1.5-fold decrease; *P* < 0.05 for all).

**Figure 1:**
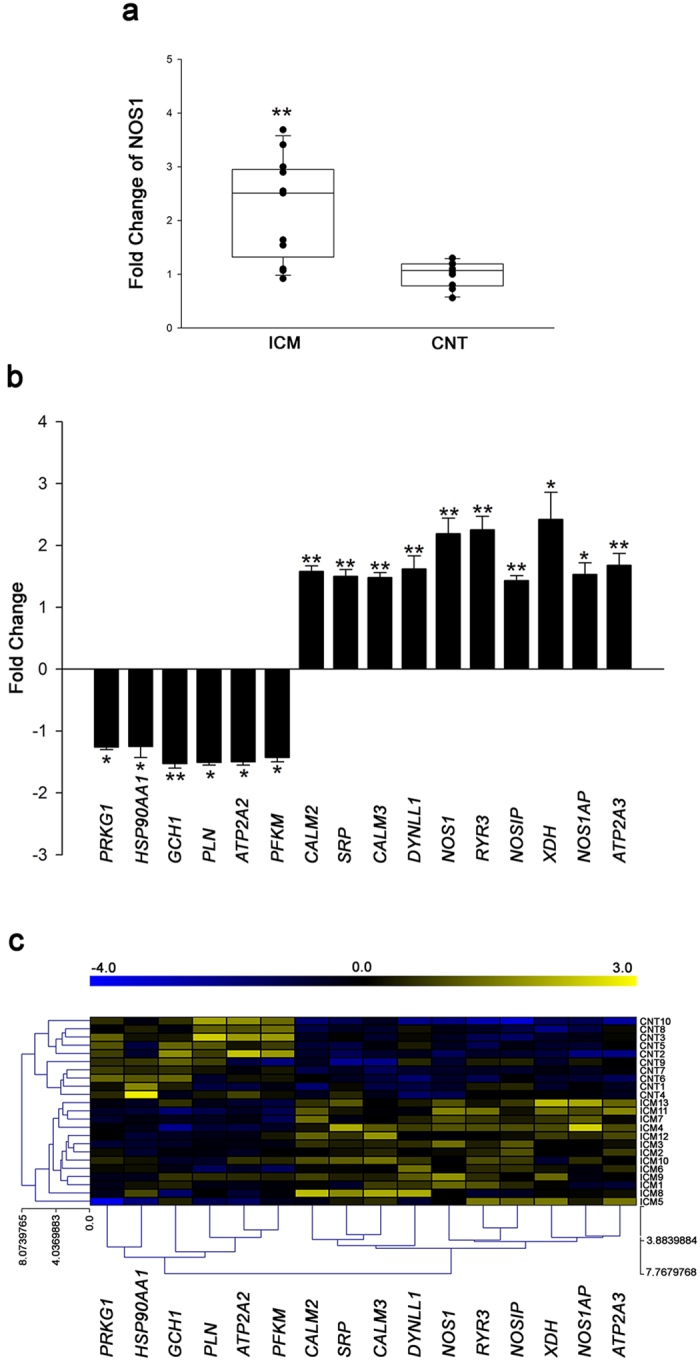
mRNA expression levels of altered NOS-related genes involved in regulating physiological function of myocyte in human ischemic hearts. (**a**) Boxplot with dots of mRNA relative expression levels of NOS1. Middle line in box represents the median, lower box bound the first quartile, upper box bound the third quartile, whiskers the 95% confidence interval of the mean. (**b**) The graph shows the values obtained by RNA-sequencing. The values from the controls were set to 1. Fold change (FC) units represent the fold induction over CNT of mRNA relative expression levels. Bars display FC ± SEM (standard error of the mean). (**c**) Heat map and hierarchical clustering based on the FC values. Heat map and hierarchical clustering analyses shows the separation of both ICM and CNT groups. Columns: genes; rows: samples. Colors depict the relative expression level of each gene; blue: lowest, yellow: highest. *P < 0.05, **P < 0.01 versus the CNT group.

We focused on 28 NOS-related genes involved in the regulation of myocardial Ca^2+^ fluxes and found significant differences in 16 NOS-related genes between the ICM samples and the CNTs. As shown in Fig. 1a and Supplementary Table S1, *NOS1* was overexpressed in the ICM samples compared to the CNTs, while we did not find similar changes in *NOS2* and *NOS3*. Important NOS-related genes such as *GCH1, HSP90AA1, ATP2A2* (gene encoding SERCA2a protein), *PLN, PFKM,* and *PRKG1* had significantly decreased mRNA levels in the ICM samples, while others such as *XDH, NOS1AP, DYNLL1, NOSIP, CALM2, CALM3, SRP, ATP2A3,* and *RyR3* were upregulated.

A heat map and hierarchical clustering were performed using MeV (v. 4.9.0) program to compare the altered genes in ICM samples with the corresponding genes in CNTs. Notably, this analysis identified two divergent gene expression profiles, showing a clear demarcation between the ICM and the CNT group (Fig. 1b).

### Differential GTP cyclohydrolase 1 and xanthine oxidoreductase protein levels in human ischemic hearts

**Figure 2:**
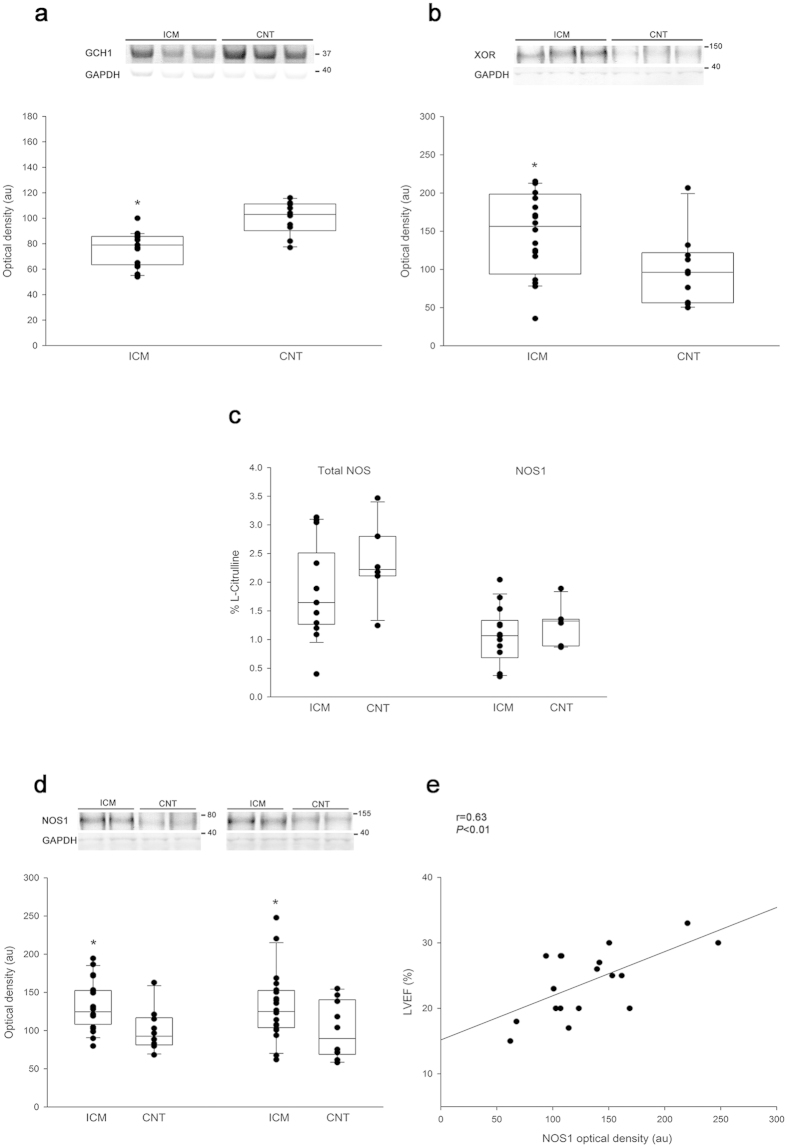
Differential GTP cyclohydrolase 1 and xanthine oxidoreductase protein levels in human ischemic hearts. Nitric oxide synthase 1 activity, levels, and its relationship with LV function. Nitric oxide synthase 1 activity, levels, and its relationship with LV function. (**a**) GTP cyclohydrolase 1 (GCH1) protein levels. (**b**) xanthine oxidoreductase (XOR) protein levels. (**c**) Total NOS and NOS1 activity in myocardial tissue from non-failing and ischemic hearts. (**d**) NOS1 protein levels. Dimeric and monomeric NOS1 determination. Values from the CNT group were set to 100. The data are expressed in optical density, arbitrary units (au). The values are normalized to GAPDH and finally to the CNT group. In the boxplots with dots the middle line in box represents the median, lower box bound the first quartile, upper box bound the third quartile, whiskers the 95% confidence interval of the mean. (**e**) Correlation between NOS1 protein levels and left ventricular ejection fraction (LVEF) in ICM patients. *P < 0.05 versus the CNT group.

Using western blotting, with total heart samples increased to 30, we found changes in the protein levels of GCH1 and XOR. ICM group had lower levels of GCH1 than the CNT group (76 ± 14 au versus 100 ± 13 au with *P* < 0.05; Fig. 2a) and higher levels of XOR (147 ± 53 au versus 100 ± 47 au with *P* < 0.05; Fig. 2b).

### Nitric oxide synthase 1 activity, protein levels, and its relationship with LV function

As shown in Fig. 2c total NOS and NOS1 activity were unchanged. In addition, both dimeric and monomeric NOS1 were significantly increased in the ICM samples (132 ± 45 au versus 100 ± 37 au with *P* < 0.05 and 132 ± 32 au versus 100 ± 27 au with *P* < 0.05, respectively; Fig. 2d). No changes were observed in the dimer/monomer NOS1 ratio in the ICM group. Next, we investigated the potential relationships between the protein levels of NOS1 and the LV function and haemodynamic parameters in the ICM group. The complete parameters of LV function were available in 18 of 20 samples and cardiac index in 13 of 20 samples. Interestingly, as showed in Fig. 2d, we found a significant relationship between the LV ejection fraction and the NOS1 level (r = 0.63, *P* < 0.01). We obtained a statistical trend between this protein and cardiac index (r = 0.484, *P* = 0.094).

### Nitric oxide synthase 1 translocation in human ischemic hearts

**Figure 3:**
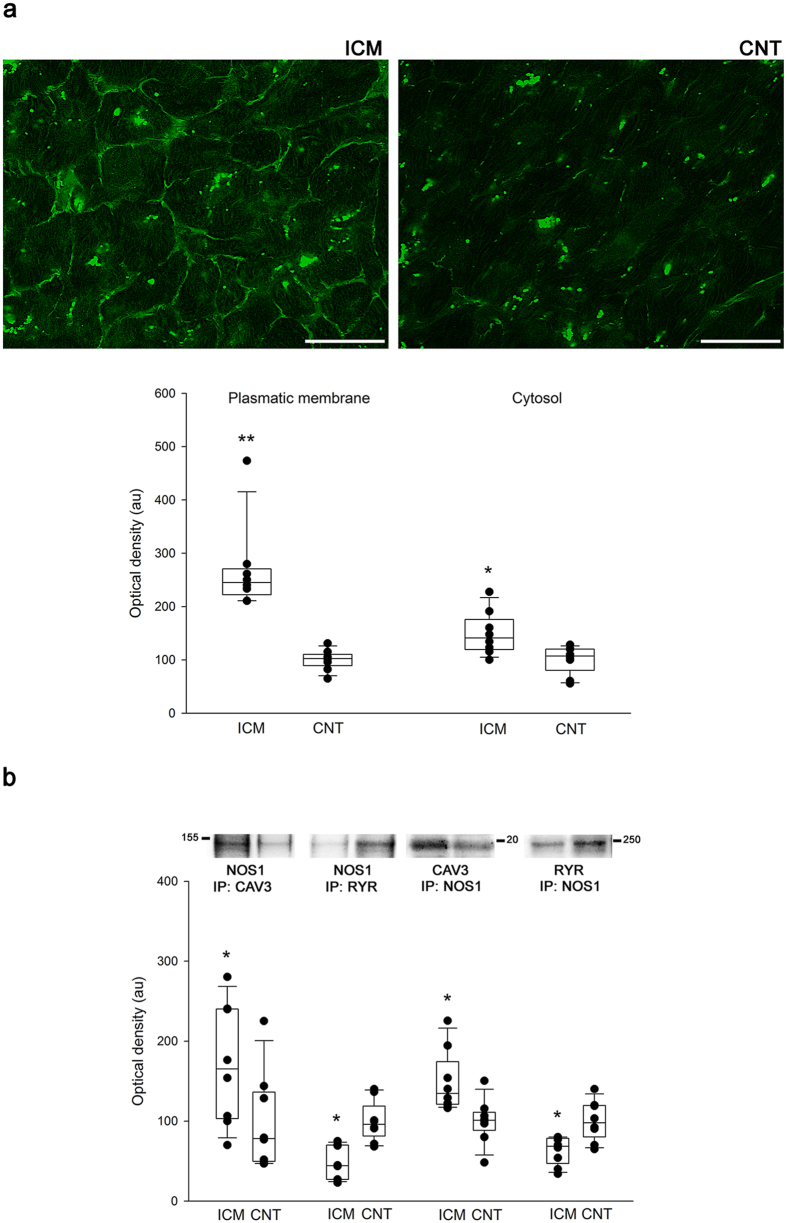
Nitric oxide synthase 1 translocation in human ischemic hearts. (**a**) The bar represents 100 μm. The green aggregated spots are lipofucsin particles (intense green in cytosol). Boxplot with dots comparing NOS1 fluorescence intensity in the membrane and cytosol in control compared to ischemic hearts. (**b**) Association between NOS1 and either caveolin 3 or ryanodine receptor. Immunoprecipitates (IP) were immunoblotted for NOS1, caveolin 3 or RyR, respectively. In the boxplots with dots the middle line in box represents the median, lower box bound the first quartile, upper box bound the third quartile, whiskers the 95% confidence interval of the mean. *P < 0.05, **P < 0.01 versus the CNT group.

Immunofluorescence experiments revealed partial translocation of NOS1 from the SR to the sarcolemma in ICM hearts (Fig. 3a). No fluorescent signal was observed when samples were only incubated with secondary antibody (not shown). In order to more specifically assess the subcellular localisation of NOS1, we studied protein-protein interactions of NOS1 with caveolin 3 (membrane protein) and also with RyR (Fig. 3b). Levels of caveolin 3-NOS1 were increased in the ICM group compared to the CNT group (171 ± 79 au versus 100 ± 62 au, *P* < 0.05). In contrast, levels of RyR-NOS1 were decreased in the ICM group (47 ± 21 au versus 100 ± 27 au, *P* < 0.05). The reverse experiment, co-immunoprecipitation of NOS1 with caveolin 3 or RyR by western blotting, confirmed these results. We found similar caveolin 3 and RyR protein levels when we compared pathological hearts with CNTs (96 ± 25 au versus 100 ± 26 au and 97 ± 11 au versus 100 ± 10 au, respectively). These findings demonstrate that a significant amount of NOS1 is translocated from the SR to the membrane, where it interacts with caveolin 3.

## Discussion

This study demonstrates the presence of important alterations in different NOS-related counterparts involved in regulation of physiological function of cardiomyocyte in cardiac tissue of ICM patients. We found relevant changes in NOS1 expression and localisation as well as in some closely related molecules involved in the regulation of its activity. No changes were observed in NOS1 activity. In addition, we found a significant relationship between NOS1 protein levels and systolic ventricular function.

Increased NOS1 levels in failing myocardium did not necessarily translate into higher nitric oxide production rates. We found a substantial increase in the gene and protein levels of NOS1 in ICM hearts in absence of change in its activity. In addition, we observed a decrease in the gene and protein levels of GCH1, the rate-limiting enzyme in BH4 biosynthesis. Thus, we obtained alterations in the enzymatic system of the main cofactor of NOS1 in human ischemic hearts. Furthermore, NOS1 is highly regulated, requiring binding of CaM, HSP90, NOSIP, and PIN (*DYNLL1* gene) for its activity, and several signalling pathways initiate NOS activity by raising intracellular Ca^2+^ concentration^11^. We found upregulation of *CALM2, CALM3, NOSIP,* and *DYNLL1*, and downregulation of *HSP90AA1* in the pathological group. Our results demonstrate that the increase found in cardiac NOS1 expression is not accompanied by an increase in its activity in ICM hearts, probably due in part to the alterations observed in the other molecules involved in the regulation of NOS1 activity. On the other hand, earlier studies on the directionality of changes in NOS2 and NOS3 isoforms in HF are contradictory^16^,^17^,^18^. These discrepancies could be due to differences in the underlying cause of development of HF. In our study, we did not observe changes in these isoforms.

The central role of cardiac SR and its Ca^2+^-handling proteins, including RyRs, SERCA2a, and phospholamban (PLB), in excitation-contraction coupling is well known^19^,^20^. NOS1 is normally located in the SR, where it associates with RyR and XOR, and where it is involved in the functions of calcium-handling molecules. The translocation of NOS1 from the SR to the sarcolemma has been reported in various animal models of HF and in human non-ischemic dilated cardiomyopathy^14^,^15^. Our results precisely demonstrate this specific subcellular change from the cytosol to the plasmatic membrane, in human hearts with ICM, where this enzyme interacts with caveolin 3. This alteration would result not only in reduced NOS1–RyR colocalisation leading to reduced RyR S-nitrosylation, but also in suppressed inhibition of XOR leading to increased O_2_^−^ production^13^. In addition, we observed upregulation of XOR in the pathological hearts. Taking into account the loss of XOR inhibition and the increase of XOR protein, we suggest these mechanisms as important contributors to myocardial redox state in agreement with the previously published studies involving rodents and non-ischemic dilated cardiomyopathy patients^21^,^22^,^23^,^24^. Moreover, the increased nitric oxide production in caveolae may enhance feedback inhibition of L-type Ca^2+^ channel and decrease local nitric oxide in the SR, thus disrupting the normal regulation of RyR2 and SERCA2a. In HF, the level and the activity of SERCA2a are decreased, contributing directly to impaired cardiac contraction and relaxation^19^,^20^. As was expected, we found downregulation of *SERCA2a* in our study. It has been reported that SERCA2a is impaired in the absence of NOS1. Taking into consideration all these changes, translocation of NOS1 from the SR to the sarcolemma may have relevant effects on myocardial contractility. Besides, we observed alterations in the expression of *RyR3* and not in that of *RyR2*; this could open an unexplored area of research on the role of this isoform in HF.

Furthermore, we found a significant relationship between NOS1 protein levels and LV ejection fraction. This result is in agreement with the previously published studies using experimental models since NOS1 has been shown to act as a major modulator of cardiac function. NOS1 in the LV myocardium of chronically infarcted animals appears to attenuate adverse LV remodeling and functional deterioration^9^ Our results provide novel evidence suggesting that NOS1 plays a crucial role in the remodeling of human ICM. A remarkable finding was the positive correlation between the level of NOS1 protein and the LV systolic function indicating that an increased NOS1 level is associated with a better ventricular performing. From these findings and taking into account the previously published literature involving experimental HF models, we infer that NOS1 may delay the development of HF due to its involvement in the myocardial response to injury in ischemic conditions. Thus, the increased levels of NOS1 could be a mechanism to compensate the loss of its activity. However, the molecular mechanisms by which the upregulation of cardiac NOS1 in absence of increase in NOS1 activity could improve the ventricular function are unknown. We can find a possible explanation in the relocation of the enzyme, possibly nitric oxide released in the sarcolemma may be affecting a different profile of proteins also involved in the regulation of ventricular function in ischemic hearts. In this sense, more studies are needed to assess this point.

Dimerisation is a requirement for catalytic activity of NOS. The dimeric form catalyzes the rate-limiting step in the synthesis of nitric oxide, while the monomeric form catalyzes the synthesis of O_2_^−^, a highly reactive oxidant species^25^. In this work, dimeric and monomeric NOS1 concentrations were higher in the ICM group; however, we did not find differences in the dimer/monomer NOS1 ratio. Notably, the dimer conformation or the distribution of NOS1 between the monomeric and dimeric forms did not change in ischemic hearts in contrast with the results of the previous studies involving other pathologic conditions^26^. In this sense, the pathophysiological relevance of the distribution of NOS1 between monomeric and dimeric forms in ICM seems questionable.

A common limitation of studies in which the cardiac tissues used are obtained from end-stage failing human hearts is that disease etiology and treatments vary considerably. However, we ensured that our study population was etiologically homogeneous.

In summary, we detected differentially expressed genes involved in the regulation of physiological function of myocyte by NOS in ventricular tissue of ICM patients. We demonstrate that the upregulation of cardiac NOS1 is not accompanied by an increase in NOS1 activity, due in part to the alterations found in molecules involved in the regulation of its activity. We observed partial translocation of NOS1 to the sarcolemma in ischemic hearts, and a direct relationship between its levels and LV ventricular function. These results suggest that NOS1 may be important in the pathophysiology of cardiac dysfunction in ischemic heart disease with a preservative role in maintaining myocardial homeostasis.

## Methods

### Collection of samples

LV tissue samples were obtained from 30 explanted human hearts: 20 from patients with ICM and 10 from non-diseased CNTs. Diagnosis of ICM was based on clinical history, Doppler echocardiography, and coronary angiography. Patients with primary valvular disease were excluded from the study. Patients were classified according to the functional criteria of the New York Heart Association (NYHA) and received medical treatment according to the guidelines of the European Society of Cardiology^27^. Clinical characteristics of patients are summarized in Table 1.

All CNTs had normal LV function (EF > 50%), as determined by Doppler echocardiography. None had any history of cardiac disease. CNT samples were obtained from non-diseased donor hearts that had been rejected for cardiac transplantation owing to size or blood type incompatibility, or due to non-availability of a suitable recipient within the requisite time period. All these donors died of either cerebrovascular or motor vehicle accidents.

Our hospital has been ranked as the national leader in heart transplantation for the third time with more than 700 transplants accomplished in the last 25 years. In accordance we acquire high-quality samples as revealed by high values (greater or equal to 9) of RNA integrity number (RIN). We have access to operating rooms during interventions and full explanted hearts in all cases. For each procedure, we choose tissue sample from the same area of the left ventricle to standardize our research methodology. In our study, all tissue samples were obtained from near the apex of the left ventricle, were maintained in 0.9% NaCl, and were preserved at 4 °C for a maximum of 4.4 ± 1.3 hours after the loss of coronary circulation. Samples were stored at −80 °C till used.

This study was approved by the Ethics Committee (Biomedical Investigation Ethics Committee of La Fe University Hospital of Valencia, Spain). Prior to tissue collection, signed informed consent was obtained from each patient. The study was conducted in accordance with the guidelines of the Declaration of Helsinki^28^.

### RNA sequencing analysis

Methods used for RNA extraction, RNAseq, computational analysis, and gene functional annotation of the RNAseq data were performed as previously described^29^. Data are included as Supplementary Information. For this analysis, LV tissue samples were obtained from 13 ICM patients and 10 non-diseased controls. The data presented in this publication have been deposited in the NCBI Gene Expression Omnibus (GEO) and can be retrieved using the GEO Series accession number GSE55296 (http://www.ncbi.nlm.nih.gov/geo/query/acc.cgi?acc=GSE55296).

### Homogenization of samples and protein determination

Twenty-five milligrams of frozen left ventricle was transferred into Lysing Matrix D tubes designed for use with the FastPrep-24 homogenizer (MP Biomedicals, USA) in a total protein extraction buffer (2% SDS, 10 mM EDTA, 6 mM Tris–HCl, pH 7.4) with protease inhibitors (25 μg/mL aprotinin and 10 μg/mL leupeptin). The homogenates were centrifuged at 16100 × *g*, and the supernatant was aliquoted. The protein content of the aliquot was determined using Peterson’s modification of the micro Lowry method with bovine serum albumin (BSA) as the standard.

### Polyacrylamide gel electrophoresis and western blot analysis

We determined the protein levels with total heart samples increased to 30. Protein samples for detecting NOS1 and XOR were separated using Tris-Acetate Midi gel electrophoresis with 3–8% polyacrylamide, and GCH1 was separated using Bis-Tris electrophoresis on 4–12% polyacrylamide gels under reducing conditions. After electrophoresis, the proteins were transferred from the gel to a PVDF membrane using the iBlot Dry Blotting System (Invitrogen Ltd, UK) for western blot analysis. The membranes were blocked overnight at 4 °C with 1% BSA in Tris buffer solution containing 0.05% Tween 20 and thereafter, were incubated for 2 hours with the primary antibody in the same buffer. The primary detection antibodies used were anti-NOS1 mouse monoclonal antibody (1:100, obtained from Santa Cruz Biotechnology, INC), anti-XOR rabbit monoclonal antibody (1:4000, obtained from Abcam, Cambridge, UK), anti-GCH1 rabbit polyclonal (1:1000, obtained from Abcam, Cambridge, UK), and anti-GAPDH mouse monoclonal antibody (1:1000, obtained from Abcam, Cambridge, UK) as a loading control.

The bands were visualized using an acid phosphatase-conjugated secondary antibody and nitro blue tetrazolium/5-bromo-4-chloro-3-indolyl phosphate (NBT/BCIP, Sigma-Aldrich, St. Louis, USA) substrate system. Finally, the bands were digitalized using an image analyzer (DNR Bio-Imagining Systems, Israel) and quantified with the GelQuant Pro (v. 12.2) program.

### Nitric oxide synthase 1 activity

Total NOS and NOS1 activity were measured using radiochemical detection of L-arginine to L-citrulline conversion, as described previously^6^. Briefly, separation of the products of L-arginine metabolism was obtained by ion exchange chromatography (Jasco Ltd.) and on-line radiochemical scintillation detection (Lablogic Systems Ltd). Recorded data were analysed using Azur software (Datalys, France). Left ventricle was homogenized ice-cold Krebs’ HEPES Buffer containing 5 μmol/L or-NOHA (to inhibit arginase activity). After centrifugation (13,000 rpm for 10 mins at 4 °C), the supernatant was then incubated for 30 mins on ice with added NOS cofactors except BH4 (i.e., 10 μmol/L FAD, 10 μmol/L FMN, 1 mmol/L NADPH), in the presence or absence of either the non-specific NOS inhibitor, L-NAME (1 mmol/L), or the NOS1-selective inhibitor SMTC (100 nmol/L), followed by 4 hours incubation at 37 °C with 3 μL of labelled^ 14^C L-arginine (Amersham Biosciences UK Ltd.). Trichloroacetic acid (10%) was then added to de-proteinate the samples, prior to centrifugation. The supernatant was placed into the auto-sampler cooled to 4 °C for chromatographic analysis. Standards of ^14^C-labelled Larginine (1 μmol/L), L-citrulline (0.1 μmol/L), and L-ornithine (0.2 μmol/L, all from Amersham Bioscience UK Ltd.) were used to determine elution time. Chromatographic peaks were integrated and expressed as a proportion of total ^14^C counts for each sample. Results were expressed as the L-NAME- or SMTC-inhibitable fraction.

### Fluorescence microscopy

Frozen muscular sections were transferred to glass slides and fixed in 4% paraformaldehyde for 15 minutes at 4 °C. Thereafter, samples were blocked with PBS containing 1% BSA for 15 minutes at room temperature. After blocking, sections were incubated for 120 minutes at room temperature with NOS1 primary antibody (described in western blot analysis) in the same buffer solution, and then with Alexa Fluor^®^ 488 conjugate secondary antibody (Invitrogen, Oregon, USA) for 60 minutes at room temperature. Finally, sections were rinsed in PBS, mounted in Vectashield conjugated 4,6-diamidino-2-phenylindole (DAPI) in order to identify the nucleus (Vector Laboratories, CA, UK), and observed with a confocal Leica TCS SP5 fluorescence microscope.

### Immunoprecipitation

For immnunoprecipitation, lysate proteins of human hearts were incubated overnight at 4 °C under gentle rotation with either caveolin 3 monoclonal antibody (obtained from Enzo Life Science, New York, USA), RyR monoclonal antibody (obtained from Thermo Scientific, Waltham, USA) or NOS1 monoclonal antibody (obtained from Santa Cruz Biotechnology, INC) at a final concentration of 5 μg/mL in 100 μL of lysis buffer. High affinity Protein A or G-conjugated agarose (obtained from Abcam, Cambridge, UK; 20 μL) were added for 3 hours at 4 °C, centrifugated, and washed three times. This was followed by resuspending the pellet with 20 μL SDS sample buffer and heating the samples to 95 °C for 5 minutes. Thereafter, samples were separated by electrophoresis and immunoblotted.

### Statistical methods

Data were expressed as mean ± standard deviation for continuous variables and as percentage for discrete variables. Significant mean differences between groups were analysed using the Mann-Whitney U test. Pearson’s correlation coefficients were calculated to determine the relationships between altered proteins and echocardiographic parameters. *P* < 0.05 was considered statistically significant. All statistical analysis was performed using the SPSS software for Windows (version 20.0; IBM SPSS Inc; Chicago. IL, USA).

## Additional Information

**How to cite this article**: Roselló-Lletí, E. *et al.* Human Ischemic Cardiomyopathy Shows Cardiac Nos1 Translocation and its Increased Levels are Related to Left Ventricular Performance. *Sci. Rep.* **6**, 24060; doi: 10.1038/srep24060 (2016).

## Supplementary Material

Supplementary Information

## Figures and Tables

**Table 1:** Clinical characteristics of patients with ischemic cardiomyopathy.

	RNAseq analysis	Protein analysis
	ICM (n = 13)	ICM (n = 20)
Age (years)	54 ± 7	55 ± 8
Gender male (%)	100	100
NYHA class	3.5 ± 0.4	3.6 ± 0.4
Prior smoking (%)	84	86
BMI (kg/m^2^)	26 ± 4	26 ± 4
Total cholesterol (mg/dL)	162 ± 41	170 ± 39
Hypercholesterolemia (%)	17	15
Prior hypertension (%)	30	33
Prior diabetes mellitus (%)	38	42
Hemoglobin (mg/mL)	14 ± 3	14 ± 2
Hematocrit (%)	41 ± 6	41 ± 5
Duration of disease (months)	45 ± 40	40 ± 37
Echo-Doppler study		
EF (%)	24 ± 4	24 ± 5
FS (%)	13 ± 2	14 ± 4
LVESD (mm)	55 ± 7	55 ± 9
LVEDD (mm)	64 ± 7	63 ± 9

Data are showed as the mean value ± SD. ICM, ischemic cardiomyopathy; NYHA, New York Heart Association; BMI, body mass index; EF, ejection fraction; FS, fractional shortening; LVESD, left ventricular end-systolic diameter; LVEDD, left ventricular end-diastolic diameter.
