# Supplementary material for: Human Ischemic Cardiomyopathy Shows Cardiac Nos1 Translocation and its Increased Levels are Related to Left Ventricular Performance
Source: Sci Rep. 2016 Apr 4;6:24060. doi: 10.1038/srep24060 (PMC4819187; doi:10.1038/srep24060)
Supplement: Supplementary Information [file srep24060-s1.pdf]

HUMAN ISCHEMIC CARDIOMYOPATHY SHOWS CARDIAC NOS1 TRANSLOCATION AND ITS INCREASED LEVELS ARE RELATED TO LEFT VENTRICULAR PERFORMANCE

Esther Roselló-Lletí[1], Ricardo Carnicer[2], Estefanía Tarazón[1], Ana Ortega[1], Carolina Gil-Cayuela[1], Francisca Lago[3], Jose Ramón González-Juanatey[3], Manuel Portolés[1], Miguel Rivera[1]

[1]Cardiocirculatory Unit, Health Institute of La Fe University Hospital (IIS La Fe), Valencia, Spain; [2]Department of Cardiovascular Medicine, University of Oxford, United Kingdom.[3]Cellular and Molecular Cardiology Research Unit, Department of Cardiology and Institute of Biomedical Research, University Clinical Hospital, Santiago de Compostela, Spain.

SUPPLEMENTARY INFORMATION

SUPPLEMENTARY METHODS

RNA extraction

Heart samples were homogenized in TRIzol® reagent in a TissueLysser LT (Qiagen, UK). All RNA extractions were performed using a PureLink™ Kit according to the manufacturer's instructions (Ambion Life Technologies, CA, USA). RNA was quantified using a NanoDrop1000 spectrophotometer (Thermo Fisher Scientific, UK), and the purity and integrity of the RNA samples were measured using an Agilent 2100 Bioanalyzer with an RNA 6000 Nano LabChip kit (Agilent Technologies, Spain). All samples showed a 260/280 ratio of >2.0 and an RNA integrity number of ≥9.

RNAseq

The RNA samples were isolated using a MicroPoly(A) Purist Kit™ (Ambion, USA). The total polyA-RNA samples were used to generate whole transcriptome libraries that were sequenced on a SOLiD 5500XL platform as per the manufacturer's recommendations (Life Technologies, CA). The amplified cDNA quality was analyzed using the Bioanalyzer 2100 DNA 1000 kit (Agilent Technologies, Spain), and the cDNA was quantified using the Qubit 2.0 Fluorometer (Invitrogen, UK). Whole transcriptome libraries were used to generate SOLiD templated beads by following the SOLiD Templated Bead Preparation guide. Bead quality was estimated based on WFA (workflow analysis) parameters. The samples were sequenced using the 50625 paired-end protocol, which generated 75 nt + 35 nt (Paired-End) + 5 nt (Barcode) sequences. Quality data were measured using the SETS software parameters (SOLiD Experimental Tracking System).

Computational analysis of RNAseq data

The initial whole transcriptome paired-end reads obtained from the sequencing were mapped against the latest version of the human genome (Version GRchr37/hg19) by using the Life Technologies mapping algorithm (http://www.lifetechnologies.com/). The aligned records were reported in the BAM/SAM format[1]. Bad quality reads (Phred score <10) were eliminated using the Picard Tools software[2].

The isoform and gene predictions were subsequently estimated using the cufflinks method[3], and the expression levels were calculated using the HTSeq software[4]. The Edge method was applied to analyze the differential expression between conditions[5]. This method relies on a Poisson model to estimate the RNAseq data variance for differential expression. We selected genes and isoforms that were calculated to exhibit $P<0.05$ and fold-change >1.5.

NOS activity

NOS activity was measured using radiochemical detection of L-arginine to L-citrulline conversion, as described previously.[6] Briefly, separation of the products of L-arginine metabolism was obtained by ion exchange chromatography (Jasco Ltd.) and on-line radiochemical scintillation detection (Lablogic Systems Ltd). Recorded data were analyzed using Azur software (Datalys, France). LV was homogenized ice-cold Krebs' HEPES Buffer containing 5 μmol/L or-NOHA (to inhibit arginase activity). After centrifugation (13,000 rpm for 10 mins at 4oC), the supernatant was then incubated for 30 mins on ice with added NOS cofactors except BH4 (i.e., 10 μmol/L FAD, 10 μmol/L FMN, 1 mmol/L NADPH), in the presence or absence of either the non-specific NOS inhibitor, L-NAME (1 mmol/L), or the NOS1-selective inhibitor SMTC (100 nmol/L), followed by 4 hours incubation at 37ºC with 3 μL of labelled ^{14}C L-arginine (Amersham Biosciences UK Ltd.). Trichloroacetic acid (10%) was then added to de-proteinate the samples, prior to centrifugation. The supernatant was

placed into the auto-sampler cooled to 4oC for chromatographic analysis. Standards of ^{14}C-labelled Larginine (1 μmol/L), L-citrulline (0.1 μmol/L), and L-ornithine (0.2 μmol/L, all from Amersham Bioscience UK Ltd.) were used to determine elution time. Chromatographic peaks were integrated and expressed as a proportion of total ^{14}C counts for each sample. Results were expressed as the L-NAME- or SMTC-inhibitable fraction.

SUPPLEMENTARY REFERENCES

1. Li, H. The Sequence Alignment/Map format and SAMtools. *Bioinformatics.* **25**, 2078-2079 (2009).
2. McKenna, A., et al. The Genome Analysis Toolkit: a MapReduce framework for analyzing next-generation DNA sequencing data. *Genome Res.* **20**, 1297-1303 (2010).
3. Trapnell, C., et al. Transcript assembly and quantification by RNASeq reveals unannotated transcripts and isoform switching during cell differentiation. *Nat Biotechnol.* 28, 511-515 (2010).
4. Anders, S. & Huber, W. Differential expression analysis for sequence count data. *Genome Biol.* **11**, R106 (2010).
5. Robinson, M.D., McCarthy, D.J., & Smyth, G.K. EdgeR: a Bioconductor package for differential expression analysis of digital gene expression data. *Bioinformatics.* **26**, 139-140 (2010).
6. de Bono, J.P., Warrick, N., Bendall, J.K., Channon, K.M. & Alp, N.J. Radiochemical HPLC detection of arginine metabolism: measurement of nitric oxide synthesis and arginase activity in vascular tissue. Nitric Oxide. **16**, 1-9 (2007).

SUPPLEMENTARY RESULTS

Supplementary Table S1. Nitric oxide synthases (NOS) and NOS1- related molecules in human ischemic cardiomyopathy.

Gene name	Description	FC±SD	p value
NOS1	nitric oxide synthase 1	2.19±0.93	*P*<0.01
NOS2	nitric oxide synthase 2	1.27±0.82	NS
NOS3	nitric oxide synthase 3	-1.07±0.44	NS
GCH1	GTP cyclohydrolase 1	-1.53±0.26	*P*<0.01
XDH	xanthine dehydrogenase	2.42±1.57	*P*<0.01
RYR1	ryanodine receptor 1	1.12±0.61	NS
RYR2	ryanodine receptor 2	-1.09±0.16	NS
RYR3	ryanodine receptor 3	2.25±0.80	*P*<0.01
PLN	phospholamban	-1.51±0.14	*P*<0.05
ATP2A1	ATPase, Ca++ transporting, cardiac muscle, fast twitch (SERCA1)	1.08±0.76	NS
ATP2A2	ATPase, Ca++ transporting, cardiac muscle, slow twitch 2 (SERCA2)	-1.50±0.16	*P*<0.05
ATP2A3	ATPase, Ca++ transporting, ubiquitous (SERCA3)	1.68±0.51	*P*<0.01
PRKG1	protein kinase, cGMP-dependent, type I	-1.26±0.15	*P*<0.05
PRKG2	protein kinase, cGMP-dependent, type II	-1.11±0.98	NS
SRP	sepiapterin reductase	1.50±0.39	*P*<0.01
NOS1AP	nitric oxide synthase 1 adaptor protein	1.53±0.69	*P*<0.05
CALM1	calmodulin 1	1.11±0.20	NS

CALM2	calmodulin 2	1.58±0.32	$P<0.01$
CALM3	calmodulin 3	1.48±0.28	$P<0.01$
CAV1	caveolin 1	-1.27±0.28	NS
CAV3	caveolin 3	1.11±0.30	NS
HSP90AA1	heat shock protein 90kDa alpha (cytosolic), class A member 1	-1.25±0.13	$P<0.05$
HSP90AB1	heat shock protein 90kDa alpha (cytosolic), class B member 1	-1.23±0.18	NS
DYNLL1	dynein, light chain, LC8-type 1	1.62±0.55	$P<0.01$
NOSIP	nitric oxide synthase interacting protein	1.43±0.21	$P<0.01$
PFKM	phosphofructokinase, muscle	-1.43±0.17	$P<0.01$
SNTA1	syntrophin, alpha 1	-1.07±0.12	NS
DMD	dystrophin	1.08±0.19	NS

Data are showed as the fold change (FC) value±standard deviation (SD)
